# Roles of Gut Bacteriophages in the Pathogenesis and Treatment of Inflammatory Bowel Disease

**DOI:** 10.3389/fcimb.2021.755650

**Published:** 2021-11-25

**Authors:** Lingling Qv, Sunbing Mao, Yongjun Li, Jia Zhang, Lanjuan Li

**Affiliations:** State Key Laboratory for Diagnosis and Treatment of Infectious Diseases, National Clinical Research Center for Infectious Diseases, National Medical Center for Infectious Diseases, Collaborative Innovation Center for Diagnosis and Treatment of Infectious Diseases, The First Affiliated Hospital, College of Medicine, Zhejiang University, Hangzhou, China

**Keywords:** inflammatory bowel disease (IBD), gut phage, immune response, pathogenesis, therapeutic target

## Abstract

Inflammatory bowel disease (IBD), including Crohn’s disease and ulcerative colitis, are chronic, relapsing intestinal inflammatory disorders. Although the molecular mechanisms governing the pathogenesis of IBD are not completely clear, the main factors are presumed to be a complex interaction between genetic predisposition, host immune response and environmental exposure, especially the intestinal microbiome. Currently, most studies have focused on the role of gut bacteria in the onset and development of IBD, whereas little attention has been paid to the enteroviruses. Among of them, viruses that infect prokaryotes, called bacteriophages (phages) occupy the majority (90%) in population. Moreover, several recent studies have reported the capability of regulating the bacterial population in the gut, and the direct and indirect influence on host immune response. The present review highlights the roles of gut phages in IBD pathogenesis and explores the potentiality of phages as a therapeutic target for IBD treatment.

## Introduction

Inflammatory bowel disease (IBD), including Crohn’s disease (CD) and ulcerative colitis (UC), are chronic disorders characterized by persistent inflammation in the gastrointestinal tract (Abraham and Cho, 2009). In recent years, the incidence of IBD has increased year by year, with a significant burden on the global economy and public health (Molodecky et al., 2012; Kaplan, 2015). Current drugs can only relieve symptoms, but can’t cure this disease. Therefore, it is urgent to uncover the specific etiology of IBD, and explore effective treatment strategy.

The specific etiology of IBD is complicated and remains unclear so far. However, researches have demonstrated that the genetic factors, the host immune system, and the environmental factors (e.g., intestinal microbiota) are associated with the initiation and development of IBD (Maloy and Powrie, 2011; Geremia et al., 2014; Ananthakrishnan et al., 2018; Turpin et al., 2018). In particular, the roles of gut microbiota in the pathogenesis of IBD has gained a lot of attention. Accumulating evidence has demonstrated that IBD is accompanied with the dysbiosis of intestinal bacteria (Ni et al., 2017; Sartor and Wu, 2017; Vemuri et al., 2017; Guan, 2019). However, except for the bacterial components, abundant viruses, especially prokaryotic viruses (phages) reside in the human gut (Reyes et al., 2010; Minot et al., 2011). More precisely, 90% of all enteroviruses are phages, and the remaining 10% are plant and animal viruses (Breitbart et al., 2003). It is estimated that the human gut contains approximately 10^15^ phages in total (Dalmasso et al., 2014; Carding et al., 2017), which outnumbering the gut bacteria (10^14^) by 10 folds (Qin et al., 2010). In addition to occupy the huge population, the gut phages can also shape the bacterial community structure by lysing and killing the host bacteria, modulate the immune response, and mediate the anti-inflammatory response (Ogilvie and Jones, 2015; Łusiak-Szelachowska et al., 2017; Gogokhia and Round, 2021). Hence, further understanding of the alterations of gut phage community, and interactions between gut phages and host immune system are helpful for elucidating the underlying molecular mechanisms of pathogenesis in IBD.

So far, the current traditional medical drugs (e.g., aminosalicylates, corticosteroids, and immunosuppressive agents) and surgical operations for IBD treatment focused mostly on the relief of clinical symptoms and the disease’s secondary effects (Anand B. Pithadia, 2011; Magro et al., 2020). More recently, with the deep understanding of microbiota-related pathogenicity in IBD development, the advent of biological agents opens up a new era for the management of IBD. Moreover, the treatment goal has also evolved from control of intestinal inflammation toward mucosal healing, including a restoration of mucosal barrier function (Mao and Hu, 2016). Apart from probiotics, prebiotics, synbiotics and fecal microbiota transplant, phage therapy has also been considered as a potential tool for microbiota modification (Oka and Sartor, 2020).

In this review, we provide an overview of the biology of phages, interactions between phages and their host bacteria, the gut phages community in healthy individuals, the alterations of gut phages in IBD patients and experimental models, and the underlying molecular mechanisms (e.g., immune and inflammatory regulation) that involved in the initiation and progression of IBD. Besides, phage-based therapeutic approaches for IBD treatment will also be discussed.

## Phage Biology and Interactions With Bacterial Host

Phages are viruses that infecting prokaryotic cells (e.g., bacteria and archaea), couldn’t reproduce without the host cellular machinery. Just like eukaryotic viruses, phages are either DNA- or RNA-based viruses, and the vast majority (96.3%) of them belong to the double-stranded DNA (dsDNA) viruses (Ackermann and Prangishvili, 2012).The host range of phage is defined as the strains or species of bacteria that a phage is able to infect (Hyman and Abedon, 2010). Most phages are only capable of infecting a narrow range of bacteria that are closely related. This specific selection depends on various factors, such as the specificity of phage’s host binding proteins, biochemical interactions during infection, presence of related prophages or particular plasmids, and bacterial phage-resistance mechanisms (Weinbauer, 2004; Diaz-Munoz and Koskella, 2014; Letarov and Kulikov, 2017).

The life cycles of phages encompass four patterns: lytic, lysogenic, chronic, and pseudolysogenic cycles (Weinbauer, 2004). In general, obligatory lytic phages (e.g., phage T1, T4 or T7) replicate through the lytic cycle. They bind to specific host-cell receptors, penetrate and infect host cell, hijack host cell’s replication and translation machinery to produce virions, produce lytic enzymes to lyse host cell, and finally release virions into the surrounding environment (Fernandes and Sao-Jose, 2018). Different from lytic phages, temperate phages (e.g., phage λ, P1 or P2) replicate through the lysogenic or lytic cycle. In a lysogenic cycle, they do not kill host cell and produce virions, but instead integrate the phage genome into the host’s chromosome or maintain as a plasmid, replicate with the bacterial host, and transmit to its progeny at each cell division (Carding et al., 2017). However, under some environmental stressors, such as DNA damage response, changes in nutrients, PH or temperature, exposure to antibiotics, hydrogen peroxide or foreign DNA, the temperate phages can be activated and switched to the lytic cycle (Howard-Varona et al., 2017).

Compared with the lytic and lysogenic cycle, the chronic cycle and pseudolysogeny remain poorly described. The chronic life cycle occurs in archaeal viruses or some filamentous phages (e.g., phage M13), which is characterized by continuously releasing the newly formed virions and affecting the growth rate of the infected host cell without causing the cell lysis (Munson-McGee et al., 2018; Stone et al., 2019). However, in a pseudolysogenic cycle, the phage genome neither integrates into the host cell’s chromosome nor propagates to produce newly virions. This phenomenon can be observed in poor nutritional conditions that limited bacterial DNA replication or protein synthesis (Cenens et al., 2013; Mirzaei and Maurice, 2017).

## Characteristics of Gut Phages in Healthy Individuals

It has been confirmed that the first colonization and initiation of intestinal microbiota begins during the delivery process (Fanaro et al., 2003; Salazar et al., 2014). Accumulating evidence suggests that the gut bacteria is relatively simple in the beginning of life, then it changes rapidly within the first few days after birth, and then ultimately becomes more diverse and stable over time (Sakata et al., 2005; Klaassens et al., 2007; Yatsunenko et al., 2012; Pilar Manrique et al., 2016). But whether gut phages follow this pattern or not?

The gut phage consists of two elements, the temperate phage located within bacterial genomes and the free virions or virus-like particles (VLPs) (Sutton and Hill, 2019).The first study describing the infant gut phage community was conducted in fresh fecal samples (collected from one week to three months of age) by Breitbart et al. in 2008 (Breitbart et al., 2008). Direct epifluorescence microscope (EFM) counts showed that the meconium (the first fecal excretion of a newborn) couldn’t detect any VLPs. However, about 10^8^ VLPs per gram wet weight of feces were detected by the end of the first week. Moreover, the following metagenomic sequencing analysis demonstrated that the identifiable sequences were dominated by dsDNA phage groups, such as Sipho-, Podo-, and Myoviruses. In addition, they also found that the diversity of the infant fecal viral community was extremely low and dynamic. This finding is consistent with previous studies, that low microbial diversity in infant guts (Magne et al., 2006; Breitbart et al., 2008). Later in 2015, another two studies also showed high dynamics of gut phages community during the first 2 and 2.5 years of life, respectively (Lim et al., 2015; Reyes et al., 2015). In addition to phages from the *Caudovirales* order (*Siphoviridae*, *Inoviridae*, *Myoviridae* and *Podoviridae* families), Lim et al. also described the presence of *Microviridae* family (single-stranded DNA genome) in the dominated phages, and a shift of phage community from *Caudovirales*-dominated to *Microviridae-*dominated composition by 24 months of age (Lim et al., 2015). Moreover, they also found that the richness and diversity of gut phages was greatest in the first 4 days of life, and then decreased with age (Lim et al., 2015).

Just like gut bacteria, the gut phage community is relatively stable in adults compared with infants (Lawrence et al., 2019). Studies have indicated that the gut phage community is dominated by temperate phages, and unique to individuals regardless of the genetic relatedness (Reyes et al., 2010). The extreme interpersonal diversity of human enteroviruses derives from two sources, i.e., persistence of a small portion of the global virome within the gut of each individual and rapid evolution of some long-term virome members (e.g., *Microviridae*) (Samuel Minot et al., 2013). Additionally, another recent research characterized the gut phage community as a mixture of three classes, including a core shared by more than 50% of individuals, another core found in 20-50% of people, and a set of phages that are rarely shared or unique to a person (Pilar Manrique et al., 2016). Furthermore, another study also reported the temporal stability and inter-individual diversity, and the predominance of virulent crAss-like (infecting bacteria of the order *Bacteroidales*) and *Microviridae* phages in the gut (Shkoporov et al., 2019). Significantly, the crAss-like phages are the most abundant viruses in the human gut, that accounting for up to 90% of the reads from human fecal viral metagenomes, and about 22% of the reads in the total metagenome (Dutilh et al., 2014; Yutin et al., 2018; Shkoporov et al., 2019). It is estimated that approximately 10^15^ phages exist in the intestine, which outnumber the commensal bacteria by 10-fold (Lozupone et al., 2012; Carding et al., 2017; Neil and Cadwell, 2018; Lin and Lin, 2019; Matijasic et al., 2020). However, about 10^8^ -10^9^ VLPs were detected in a gram of wet weight feces by direct counting with microscopy (Breitbart et al., 2008; Kim et al., 2011; Hoyles et al., 2014).

In general, current methods for studying the gut virome are dependent on direct observation and counting of VLPs by using EFM and transmission electron microscopy (TEM), isolation of individual phages infecting specific host bacteria strains by culturing, and the newly booming high-throughput metagenomic sequencing and bioinformatics technology (Shkoporov and Hill, 2019). Given that most of the viral metagenomic data can’t be found in the public databases, studies for the gut phage composition and function are just beginning (Reyes et al., 2012; Aggarwala et al., 2017).

## Roles of Gut Phages in IBD Pathogenesis

As described above, the gut phage community maintains dynamic equilibrium under normal physiological conditions, whereas this balance can be disturbed by various factors, such as diet, lifestyle modification or pathological status (Minot et al., 2011; Coughlan et al., 2021; Marongiu et al., 2021). Advancing evidence has demonstrated that dysbiosis of gut phages may stimulate the development or aggravate the course of diseases, such as periodontal disease, Parkinson’s disease, type 2 diabetes, cancer or gastrointestinal disease (Azeredo et al., 2016; Manrique et al., 2017; Ma et al., 2018; Tetz et al., 2018; Santiago-Rodriguez and Hollister, 2019; Maronek et al., 2020). Up to now, a number of studies have been conducted in clinical samples and experimental models to demonstrate the relationship between gut phage homeostasis and the development and progression of IBD (Norman et al., 2015; Duerkop et al., 2018; Clooney et al., 2019). According to their experimental results, we concluded that gut phages may contribute to IBD pathogenesis through the following three pathways: alteration of gut phage diversity, regulation of gut bacterial population, and modulation of pro-inflammatory action and local immune response.

### Alterations of Gut Phage Community in IBD Patients and Experimental Models

Different from bacteria or fungi, there is no universal marker gene that can be utilized to identify viruses, and the majority of obtained sequences do not exist in publicly available databases (Roux et al., 2015; Krishnamurthy and Wang, 2017). As for the changes of enteric phage community, most current studies are based on metagenomic sequencing of fecal samples and intestinal biopsies. Differences have been found between healthy individuals and IBD patients or experimental models (**Table 1**).

**Table 1:** Overview of gut phage community alterations in IBD patients and animal models.

Subjects	Samples	Results	Reference
CD patients (n=19)	Biopsies	More VLPs in CD patients than healthy individuals on the mucosal level. Less VLPs in CD ulcerated mucosa than non-ulcerated areas.	(Lepage et al., 2008)
UC patients (n=91)	Rectal mucosa	Increased abundance, but decreased diversity, richness and evenness of *Caudovirales* phages in UC mucosa. More abundant *Escherichia* phage and *Enterobacteria* phage in UC mucosa.	(Zuo et al., 2019)
CD patients (n=6) Pediatric	Ileal biopsies; colonic biopsies; gut wash samples	Increased abundance of Phage composition in CD patients than control individuals. The largest proportion of sequences were *Bacteroides* phage B40-8 and phage B124-14.	(Wagner et al., 2013)
CD patients (n=5) Pediatric	Colonic wash samples	The mucosal-luminal interface virome is subject specific.	(Yan et al., 2020)
CD patients (n=20) at different stage	Stool samples; Biopsies	Increased abundance of Phage composition in CD patients than control individuals. Phages in fecal samples were 3-fold more than in biopsies. *Alteromonadales* and *Clostridiales* phages were increased in CD subjects.	(Perez-Brocal et al., 2015)
IBD patients (n=10)	Colonic biopsies	The majority of DNA Viruses within the virome were phages. Nearly half of the phages were associated with bacterial strains identified in the colon samples.	(Wang et al., 2015)
IBD patients (n=54) VEO	Stool samples	The VEO-IBD subjects have a higher ratio of *Caudovirales* versus *Microviridae* compared to healthy controls.	(Liang et al., 2020)
CD patients (n=18); UC patients (n=42)	Stool samples	A significant expansion of *Caudovirales* phages in IBD patients, and the virome of CD and UC patients were disease- and cohort- specific.	(Norman et al., 2015)
CD patients (n=27); UC patients (n=42)	Fecal sample	A healthy core of virulent phages is replaced by temperate phages in IBD.	(Clooney et al., 2019)
CD patients (n=7); UC patients (n=5) Pediatric	Fecal samples	The relative abundance of *Caudovirales* was greater than that of *Microviridae* phages in both IBD and healthy controls. *Caudovirales* phages were more abundant in CD than UC but not controls. The richness of viral strains in *Microviridae* but not *Caudovirales* was higher in controls than CD but not UC cases.	(Fernandes et al., 2019)
CD patients(n=65) UC patients (n=38)	Stool samples	Phage community compositions were highly specific to each individual. Different abundance of temperate phages was identified between IBD and non-IBD patients. In active UC patients, temperate phages infecting *Bateroides uniformis* and *Bacteroides thetaiotaomicron* were over-represented in comparison with non-IBD patients.	(Nishiyama et al., 2020)
C57BL/6 mice (n=3)	Fecal samples	A decrease in phage community diversity, and an expansion of subsets of phages in animals with colitis. Abundance of *Clostridials* phages decreased during colitis.	(Duerkop et al., 2018)

#### IBD Patients

Dysbiosis of intestinal bacteria has been implicated in the initiation and deterioration of IBD, but what is the origin of dysbiosis? Phages outnumber bacteria by a factor of 10, and exert a strong influence on bacterial diversity and population structure. In order to ascertain whether bacterial imbalance in IBD is related to gut phages, Lepage et al. conducted the first study in 2008 to measure the total viral community in CD patients (Lepage et al., 2008). Biopsy samples were obtained to detect VLPs in the mucosa by using EFM and TEM. Viral abundance was compared between healthy individuals and patients with CD, and also between the ulcerated and non-ulcerated mucosa of these patients. They found that CD patients harbored significantly more VLPs than healthy individuals (2.9×10^9^ *vs.* 1.2×10^8^ VLPs/biopsy), whereas CD ulcerated mucosa contained less VLPs than non-ulcerated mucosa (2.1×10^9^ *vs.* 4.1×10^9^ VLPs/biopsy) (Lepage et al., 2008). More recently, another study described the mucosa virus in patients with UC. Results showed a high abundance of *Caudovirales* phages, but decreased diversity, richness and eveness of mucosa *Caudovirales* in UC patients compared with healthy controls (Zuo et al., 2019). Moreover, they also found that abundance of *Escherichia* phage and *Enterobacteria* phage was significantly higher in the mucosa of UC patients than heathy controls.

In addition to changes of phage population on the mucosa, alterations in fecal samples and colonic tissues between IBD patients and healthy controls or between different disease types (CD and UC) also have been reported. A metagenomics analysis of gut tissue and wash samples has characterized a large abundance of phages when compared with pediatric CD patients and control individuals (Wagner et al., 2013). Moreover, they also found that the largest proportions of sequences were *Bacteroides* phage B10-8 and phage B124-14, and the *Mycobacterium* phage composition was different in ileum tissue samples between CD patients and controls (Wagner et al., 2013). A recent virome sequencing study conducted with the proximal and distal colonic wash samples from pediatric CD patients demonstrated high interpatient diversity and low but significant intra-patient variation between different sites (Yan et al., 2020). In another study, the differences of bacterial and viral communities in different type of samples from adult CD patients at different stages were investigated (Perez-Brocal et al., 2015). They found that phages were more abundant in feces (3 folds) than in biopsies, and the bacterial community reflects the disease status of individuals more accurately than the viral communities. Moreover, numerous viral biomarkers specifically associated with CD disease were identified, that phages infecting bacterial orders *Alteromonadales* and *Clostridiales*, including bacterial species *Clostridium acetobutylicum* and *Retroviridae* family were increased in subjects with CD (Perez-Brocal et al., 2015). Another metagenomics analysis of colonic biopsies has demonstrated that nearly half of the phages were associated with bacterial strains identified in the colon samples (Wang et al., 2015). Additionally, a recent study has investigated the dynamics of stool virome in very early onset (VEO) IBD, which is defined as onset of IBD before 6 years of age (Liang et al., 2020). Results showed that there is no significant different of total number of VLPs between VEO-IBD and healthy controls, but the VEO-IBD subjects exhibit a higher ratio of *Caudovirales* versus *Microviridae* compared to healthy controls (Liang et al., 2020).

As mentioned above, differences of gut phages population between CD and UC patients have also been investigated. A metagenomics analysis of stool samples from CD and UC patients demonstrated that *Caudovirales* phages significantly increased in IBD patients compared with healthy cohorts. Moreover, the gut phage community of CD and UC patients were disease- and cohort-specific (Norman et al., 2015). Later, Clooney et al. reanalyzed the above published dataset of healthy and IBD gut virome. Evidence has been found to prove that a healthy core of virulent phages is replaced by temperate phages in CD patients (Clooney et al., 2019). In another study, Fernandes et al. investigated the fecal virome in children with CD, UC and age-matched healthy controls. Results indicated that the relative abundance of *Caudovirales* was greater than that of *Microviridae* phages in both IBD and healthy controls, and the *Caudovirales* phages were more abundant in CD than UC (p=0.05) but not controls, and the richness of viral strains in *Microvioridae* but not *Caudovirales* was higher in controls than CD (p=0.05) but not UC cases (Fernandes et al., 2019). Another recent study demonstrated the ecological structure of the human gut temperate phage community by using publicly available whole-metagenome shotgun sequencing data (Integrative, 2014; Lloyd-Price et al., 2019; Nishiyama et al., 2020). Consistent with previous reports, they found the compositions of temperate phage community were highly specific to each individual and the abundance was different between IBD patients and non-IBD patients. In addition, temperate phages infecting *Bacteroides uniformis* and *Bacteroides thetaiotaomicron* were over-represented in active UC patients, whereas their hosts were under-represented in comparison with non-IBD patients (Nishiyama et al., 2020).

#### Animal Experiments

Apart from researches conducted in IBD patients, animal experiments have also been an important pathway to explore the roles of enteric phages in IBD pathogenesis. A recent metagenomics study indicated that the intestinal phage population altered and transited from an ordered state to a stochastic dysbiosis in a mice model with colitis (Duerkop et al., 2018). Interestingly, these alterations are similar to those observed in human IBD patients (Norman et al., 2015). Additionally, they observed a decrease in phage community diversity, an expansion of subsets of phages and a decrease of certain phages number (e.g., *Clostridiales* phages) during colitis (Duerkop et al., 2018).

**Figure 1:**
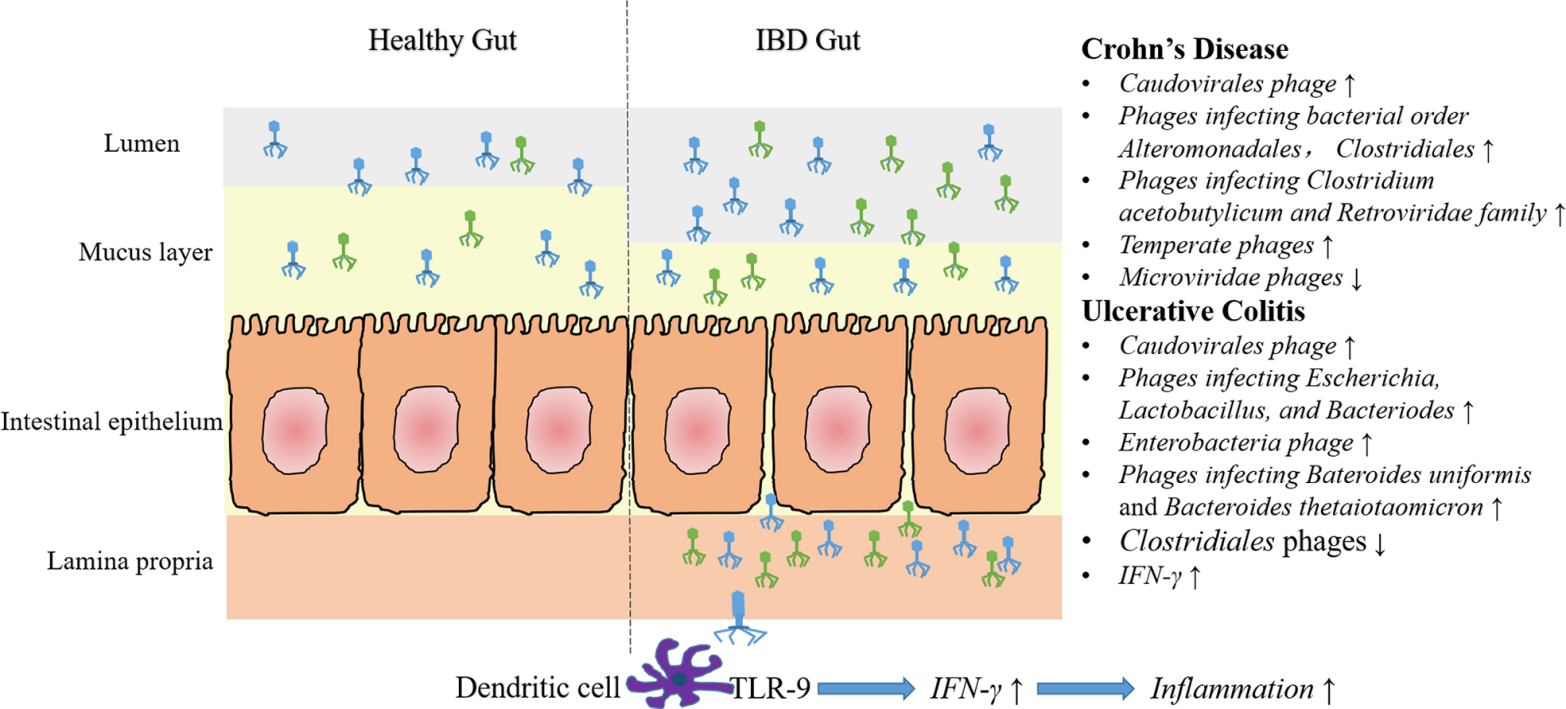
Characteristics of gut phage community and phage associated immune modulation in IBD. Compared with healthy gut, more *caudovirales* phages are found in the intestinal lumen and mucus layer. Moreover, some specific phage community changes have been demonstrated in CD and UC. In CD, an increased abundance of temperate phages and phages infecting bacterial order *Alteromonadales*, *Clostridiales*, *Retroviridae* family and *Clostridium acetobutylicum*, and a decreased abundance of *Microviridae* phages have been reported. In UC, numbers of phages infecting *Escherichia*, *Lactobacillus* and *Bacteriodes* (e.g., *Bacteroides uniformis* and *Bacteroides thetaiotaomicron*) and *Enterobacteria* phages increased, whereas *Clostridiales* phages decreased. It should be noted, however, some of the above results are just concluded from a single experiment, and that can’t be compared with each other, due to different samples and methods are used. Additionally, impaired epithelial barrier in IBD lead to increased intestinal permeability, that lead to the migration of many phage particles into the lamina propria or even the circulation. *In vitro* experiments showed that recognition of phage DNA *via* TLR9 on dendritic cells stimulated the production of IFN-γ, thereby aggravated intestinal inflammation.

In conclusion, current researches are focused on describing fecal and mucosal phage communities by using metagenomic sequencing and bioinformatic analysis. Most of the studies demonstrated the increased abundance and decreased diversity of *Caudovirales* phages both in CD and UC in comparison with health controls. However, another recent study showed no significant difference of gut phage number between IBD and healthy controls (Liang et al., 2020). Some of the experiments also showed alterations of certain phages, such as increased *Escherichia* phages and *Enterobacteria* phages in UC mucosa, decreased *Clostridials* phages during colitis, and increased *Alteromonadales* and *Clostridiales* phages in CD subjects (**Figure 1**).

### Phages Affect IBD Through Bacterial Modulation

Virulent phages that are capable of lysing host bacteria were commonly found in the gut of patients with IBD (Clooney et al., 2019). It has been shown that phage invasion affected host bacteria, resulting in changes in bacterial abundance of specific species (Reyes et al., 2013). Researches have reported the dysbiosis of gut bacteria both in CD and UC patients, with a decreased diversity, expanded potentially pathogenic *proteobacteria* (e.g., *E. coli*, *Fusobacteria*) and reduced potentially protective *Firmicutes* (e.g., *Faecalibacterium prausnitzii*, *Rumininococci* and *Clostridium clusters* IV and XIVa) (Frank et al., 2007; Hoarau et al., 2016; Lloyd-Price et al., 2019). Nishiyama et al. have demonstrated an increased abundance of phages infecting *Bacteroides uniformis* and *Bacteroides thetaiotaomicron*, and a decreased abundance of their host bacteria (Nishiyama et al., 2020). Combining the above two information, we can infer that there is a certain relationship between the phage number and its host bacteria population. Germ-free (GF) animals, without any microbial colonization in their intestines, are perfect model systems for gut microbiota related studies (Uzbay, 2019; Qv et al., 2020). A recent study investigated in GF mice models showed that phage predation directly impacts susceptible bacteria, leading to cascading effects on other bacterial species, with consequences on the gut metabolome (Hsu et al., 2019).

Except for virulent phages, temperate phages can also influence bacterial fitness and diversity (Brussow et al., 2004). Phages substantially contribute to the genetic variability of bacteria by horizon gene transfer and increasing of the mutation rate (Abeles and Pride, 2014; Janka Babickova, 2015). Prophages carrying genes encoding antibiotic resistance may provide evolutionary advantages to pathogenic or probiotic bacteria. Besides, stress-induced activation of a prophage dormant in commensal or pathogenic bacteria might lead to the activation of its lytic cycle, thereby reduce the amount of the host bacteria. A metagenomic study showed a higher prevalence or abundance of phages infecting *Faecalibacterium prausnitzii* in stools of IBD patients than in those of healthy controls (Cornuault et al., 2018). Since less *Faecalibacterium prausnitzii* has been reported in IBD patients, they suggested that phages could trigger or aggravate *Faecalibacterium prausnitzii* depletion. But how or whether these phages being activated into lytic cycle in patients are not being described.

Overall, the current evidence is still limited, how phages directly or indirectly impact bacterial communities are barely unclear and speculatively feasible. A recent review also proposed a theoretical model of temperate phages in mediating the gut bacteria community (Lin et al., 2019). Further experimental studies are needed to reveal the complicated phage-bacteria interactions in IBD.

### Phages Affect IBD Through Immune Modulation

It is well acknowledged that IBD is associated with the imbalance of immune response and inflammatory reaction. Studies have showed a relief of enteric viruses (rotavirus) in gut inflammation *via* toll-like receptor (TLR) 3 and TLR7-mediated interferon-β (IFN-β) production (Yang et al., 2016). While phages do not infect mammalian cells, several studies have demonstrated the direct and indirect influence of these prokaryotic viruses on host immune system (Hodyra-Stefaniak et al., 2015; Bollyky and Secor, 2019; Gogokhia and Round, 2021). Indeed, phages have been suggested to be a key element that helps to shape innate, humoral and cell-mediated immunity (Duerkop and Hooper, 2013).

First, phages act as a prominent defender of the mucosal barrier against bacteria. *In vitro* studies have showed that phages can adhere to mucus layer, thereby reduced microbial colonization and pathology. The adherence of phages was mediated by interactions between displayed immunoglobulin (Ig)-like domains of phage capsid proteins and glycan residues, such as those in mucin glycoprotein (Barr et al., 2013a).

In addition to provide a non-host-derived immunity, phages can also provide an acquired antimicrobial immunity, and help to control local inflammatory and autoimmune reactions in the gut (Górski and Weber-Dabrowska, 2005; Barr et al., 2013b). A recent study showed that both induction of innate and adaptive immunity was upregulated in phage-treated animals (Gogokhia et al., 2019). Results showed that the percentage of CD4+ and CD8+ T cells in the mesenteric lymph nodes (MLNs) were significantly increased. Besides, the proportions and numbers of CD4+ T cells within the Peyer’s patches (PP) were higher in GF mice that treated with phages compared with GF mice (Gogokhia et al., 2019). And *in vitro* experiments suggested that dendritic cell recognition of phage DNA can stimulate IFN-γ production through a TLR-9 dependent pathway, which functioned to exacerbate the intestinal inflammation and contribute to disease severity (Gogokhia et al., 2019).

Last but not least, just like bacteria, phages can pass the intestinal wall and migrate to lymph, peripheral blood and internal organs, and then directly modulate the host immune system (Górski et al., 2006). Besides, results have demonstrated that bacterial translocation from the gut to tissue induces inflammation and development of metabolic disease (Burcelin, 2016). Phages could limit bacterial translocation by directly eliminating sensitive bacteria, thereby indirectly inhibit gut inflammation that caused by bacterial translocation. Moreover, phages may downregulate gut immune cells such as dendritic cells and prevent pro-inflammatory action of these cells (Górski et al., 2012; Górski et al., 2016).

In conclusion, the current available data concerning the influence of gut phages on IBD by immune modulation is limited. The idea we came up above is based on the immune disorders in IBD and the regulation of immune response showed in phages. Further studies focused on the interactions between gut phages and immune response in IBD patients or animal models are urgently needed.

## Use of Phages for IBD Treatment

Due to the relapsing inflammatory disorders in IBD patients, controlling of intestinal inflammatory is the primary target of treatment. Currently, traditional therapies includes 5-aminosalicylic acid derivatives (e.g., sulfasalazine and mesalazine), corticosteroides (e.g., prednisone, hydrocortisone, budesonide, prednisolone, dexamethasone) and immunosuppressive agents (e.g., azathioprine, methotrexate, mycophenolate, cyclosporine, tacrolimus, 6-mercaptopurine) (Moja et al., 2015; Wang et al., 2016; Matsumoto et al., 2016; Bots et al., 2018; Sokollik et al., 2018; Damiao et al., 2019; Nielsen et al., 2020; Antunes et al., 2021). Although these therapies have been proved effective in IBD patients, severe adverse events with impaired quality of life can’t be ignored (Quezada et al., 2018). Recent compelling evidence has demonstrated the crucial roles of resident microbiota in driving immune dysfunction and inflammation in IBD, thereby microbial-targeted therapies are being studied for IBD treatment (Oka and Sartor, 2020). Probiotics, prebiotics and fecal microbiota transplant are commonly used for microbiota-targeted therapies, which have been proved to be safe and potentially effective for correcting the dysregulated immune response (Derwa et al., 2017; Laurell and Sjoberg, 2017; Gagliardi et al., 2018; Zuo and Ng, 2018; Cohen et al., 2019). Besides, phage therapy, as another effective method for restoring gut microbiome homeostasis (Dixit et al., 2021), has also been proposed as a therapeutic target for IBD treatment.

Indeed, phages has long been used as a therapy to treat bacterial infectious diseases (e.g., cholera and bacillary dysentery), due to their function of lysing host bacteria (Summers, 1993; Ho, 2001). In particular, with the increasing emergence of multi-drug resistance bacteria, phages are being explored in targeting and killing specific infectious bacteria that associated with intestinal infection, such as *Clostridioides difficile* in colitis (Park et al., 2019) and *Fusobacterium nucleatum* in colorectal cancer (Machuca et al., 2010). In addition to their antibacterial activity, current phage therapy also focused on the immunomodulating properties.

Currently, phage therapy used for IBD treatment mainly targeted to the adherent invasive *Escherichia coli* (AIEC). Compared with healthy individuals, the AIEC strains were much more common in CD patients (Lamps et al., 2003). Moreover, studies have suggested the involvement of AIEC strain in maintaining intestinal inflammation in IBD (Sartor and Wu, 2017; Carolina Palmela et al., 2018; Chervy et al., 2020). Several researches have showed the effectiveness of phages in reducing intestinal *E. coli* colonization (Vahedi et al., 2018; Yu et al., 2018). Vahedi and his colleagues isolated specific phages against enteropathogenic *E. coli* from hospital sewage, and proved that single dose of phage cocktail (2×10^9^ PFU/mL) was able to control the bacteria infection (Vahedi et al., 2018). A recent study has tested phage therapy against AIEC strain in a dextran sulfate sodium (DSS) induced colitis mouse model (Galtier et al., 2017). Results showed that a single day of oral treatment with phage cocktail significantly decreased the colonization of AIEC strain LF82, with reduced symptoms over a 2-week period. Additionally, in a double-blinded, placebo-controlled crossover trial, administration of a commercial cocktail of *E. coli*-targeting phages for 28 days selectively reduced the fecal *E. coli* loads without globally disrupt the gut microbiota community and increased anti-inflammatory cytokines IL-4 (Febvre et al., 2019).

In addition to the *in vivo* animal models, *in vitro* models also play vital roles in testing the therapeutic activity of gut phages. A recent study has developed an intestinal epithelium model able to produce mucus by co-culturing Caco-2 and HT29-MTX (4:1 ratio) cells (Nunez-Sanchez et al., 2020). This mammalian cell model will enable a better understanding of phage-bacteria interactions and the protective effects of phage therapy.

Collectively, researches about the efficacy of phage therapy in IBD treatment is scarce, either in clinical trials or animal experiments. More further studies are needed to clarity the ability of phages in modulate intestinal microbiota balance and immune response. Although the purified formulation of phages has been proved to be non-toxic (Divya Ganeshan and Hosseinidoust, 2019; Dixit et al., 2021), the safety of phage therapy still needed to be taken into consideration. Besides, researchers have also suggested the timing of administration of phage therapy as a challenge in the future studies (Janka Babickova, 2015).

## Conclusions and Perspectives

As a widespread and incurable enteric disease, it is urgent and meaningful to reveal the pathogenic mechanisms of IBD. Studies that exploring the roles of gut microbiome in IBD pathogenesis should not focus solely on the bacterial composition, but neglect the enteroviruses, especially gut phages, which are huge number and closely related to the bacteria. The recent fast-growing in high-throughput sequencing and bioinformatics technologies has enabled major advances in human gut phageome researches. Current scattered researches have uncovered an increased abundance and decreased diversity of *caudovirales* phages in IBD patients or experimental models in comparison with healthy individuals. However, the majority of identified phage sequences are not yet taxonomically classified, that is to say annotated sequences do not exist in the public databases (Dalmasso et al., 2014; Shkoporov and Hill, 2019). Therefore, insights based on viral taxonomy require careful consideration. In the mucosa, CD patients were found to have more phages than healthy individuals, whereas the ulcerated mucosa had fewer phages than unaffected mucosa (Lepage et al., 2008). This paradoxical result may be caused by the incomplete databases. Moreover, methods that used in existing studies vary, that can impede our ability to compare the outputs of different studies. To be noted, whether an altered phageome is the consequence or the cause of IBD is currently not fully understood. Besides, the underlying mechanisms of phages affecting IBD through bacterial modulation and immune regulation still needs more experimental evidence.

Limited understanding of phage biology and disease microbiology suppressed the usage of phage therapy. Current phage therapy for IBD treatment mainly targeted the AIEC strain. The discovery of gut bacteria that directly linked to IBD development will accelerate the application of phage therapy. Besides, the efficacy of phage therapy should also pay attention to the influence of phages on immune response apart from the antibacterial properties.

In conclusion, many key information about the human gut phageome, phage-bacteria interactions, and phage-host interactions remains unclear. In the future, further optimization of techniques for phage isolation and identification may be required. Additionally, more *in vivo* and *in vitro* studies are needed to elucidate the roles of gut phages in IBD development and treatment.

## Author Contributions

LQ researched the topics and drafted the manuscript. SM, YL, and JZ helped to search the reference during the preparation of the manuscript. LQ and LL revised the manuscript for publication. All authors read and approved the final manuscript.

## Funding

This research was supported by the Independent Project Fund of the State Key Laboratory for Diagnosis and Treatment of Infectious Diseases (2020) and grants from the National Natural Science Foundation of China (81790631 and 81330011).

## Conflict of Interest

The authors declare that the research was conducted in the absence of any commercial or financial relationships that could be construed as a potential conflict of interest.

## Publisher’s Note

All claims expressed in this article are solely those of the authors and do not necessarily represent those of their affiliated organizations, or those of the publisher, the editors and the reviewers. Any product that may be evaluated in this article, or claim that may be made by its manufacturer, is not guaranteed or endorsed by the publisher.
